# Evaluation of alternative blood culture systems in detecting pathogenic bacteria: a multi-site, observational prospective study

**DOI:** 10.1016/j.lanwpc.2026.101806

**Published:** 2026-02-02

**Authors:** Yanyan Hu, Yinfei Fang, Zelin Yan, Zhiqiang Zhu, Qiaoling Sun, Sipei Wang, Yanyan Jiang, Xinhua Qiang, Chang Cai, Yanyan Zhu, Yongjun Ma, Lihong Bu, Wenzi Bi, Xiaoping Xia, Lingbin Shu, Yangxiao Zhou, Yunxiang Cai, Hongwei Zhou, Jiachang Cai, Hanqiang Miao, Lin Huang, Yuqing Zhou, Shaolin Wang, Rong Zhang, Timothy R. Walsh

**Affiliations:** a Department of Clinical Laboratory, Second Affiliated Hospital of Zhejiang University, School of Medicine, Hangzhou, China; b Department of Clinical Laboratory, Affiliated Jinhua Hospital, Zhejiang University School of Medicine, Jinhua, Zhejiang, China; c Department of Clinical Laboratory, The Fourth Affiliated Hospital, Zhejiang University School of Medicine, Yiwu, Zhejiang, China; d Department of Laboratory Medicine, The First Affiliated Hospital, Zhejiang University School of Medicine, Hangzhou, China; e Department of Clinical Laboratory, Wenzhou Medical University Affiliated Dongyang Hospital, Dongyang, Zhejiang, China; f Medical Laboratory Center, Tongxiang First People's Hospital, Zhejiang, China; g Department of Clinical Laboratory, The First People's Hospital of Huzhou, Huzhou, China; h Sanya Institute of Nanjing Agricultural University, Sanya, China; i Ineos Oxford Institute for Antimicrobial Research, Department of Biology, Oxford, UK; j College of Veterinary Medicine, China Agricultural University, Beijing, China

**Keywords:** Sepsis, Blood culture, Time to positivity, Low- and middle- income countries

## Abstract

**Background:**

Sepsis, is a severe, life-threatening response to infection with rising incidence and significant mortality. Although blood culture (BC) systems are critical for sepsis diagnosis and the detection of pathogens bacteria, their high cost limits their use in low-income regions. This study evaluates the performance and affordability of bottles with gold-standard systems.

**Methods:**

From September 2023 to June 2024, we conducted a nine-month evaluation of alternative BC instruments (Meihua Medical [MH] and DL-Biotech [DL]) and BC bottles (BCBs) against bioMérieux and Becton Dickinson (BD) across five tertiary hospitals in Zhejiang province, China. Before processing clinical samples, we examined the performance of BCBs and instruments including time to positivity (TTP) and sensitivity after spiking bottles with *Escherichia coli* and ESKAPE bacteria. Over the course of the study, a total of 37,480 BCBs were assessed.

**Findings:**

DL and MH instruments performed well in TTP across commonly isolated bacteria. In the non-inferiority observational study, we compared instruments between MH versus BD, DL versus BD, MH versus bioMérieux, and DL versus bioMérieux, giving concordance rates of 97.2%, 97.7%, 98.0%, and 96.5%, respectively. However, when comparing sensitivity between MH and BD, and MH and bioMérieux, the number of detected bacterial isolates was 163 versus 122, and 116 versus 108, respectively. When comparing BCBs, MH versus BD bottles, and MH versus bioMérieux bottles, gave 209 and 193, and 205 and 200 positive growths, respectively. However, MH was less effective than BD in detecting anaerobic bacteria. DL had a lower overall detection rate compared to bioMérieux (130 versus 199) but exhibited similar TTP for *E. coli* and ESKAPE pathogens, except for *Enterococcus* spp., where DL demonstrated a longer TTP.

**Interpretation:**

Our evaluation of DL and MH BC systems suggests that both are comparable to BD, and bioMérieux. Their performance of alternative BC instruments and bottles, suggest that BC alternatives should be considered in providing clinical support for low- and middle-income countries, and augment appropriate antimicrobial stewardship.

**Funding:**

This study was funded by the 10.13039/501100012166National Key Research and Development Program of China (No. 2022YFD1800400) and the Theme Based Research Scheme (T11-104/22-R) from the Research Grant Council of the Government of Hong Kong SAR.

**Research in context**

**Evidence before this study**

On October 22nd 2024, we searched PubMed using terms “comparison of blood culture bottles” and “comparison of blood culture instruments” from 01.01.2014 onwards which resulted in 274 hits. After scrutinising all abstracts and including those studies directly comparing blood culture (BC) bottles and or/instruments either using simulated (spiked samples) and∖or observational clinical trials, 15 studies were of relevance. Of these 15, only one study compared new alternatives to the “gold standard” bottles/systems of Becton Dickinson (BD) and bioMérieux. Furthermore, there were no comparative analysis of alternative systems using a prospective observational clinical trial.

**Added value of this study**

To our knowledge, this is the first study to prospectively evaluate and compare alternative blood culture systems against “gold standards” in clinical settings. Our findings bridge a gap in the literature by providing detailed comparisons of alternative systems such as Meihua Medical, and Zhuhai DL-Biotech blood culture bottles, suggesting potential advantages in regions such as LMICs when affordability and diagnostic sustainability are key concerns.

**Implications of all the available evidence**

Alternative blood culture systems from DL-Biotech and Meihua Medical offer comparable performance to international brands e.g. Becton Dickinson and bioMérieux, in particular Meihua Medical often out-performed both Becton Dickinson and bioMérieux. By reducing running costs and providing enhanced access, these systems have the potential to significantly improve healthcare delivery in low-resource settings where the price of the bottles and instruments are a fraction of the current market prices. Implementing such technologies could drive substantial advancements in global health equity, bolstering laboratory capacity, significantly reducing inappropriate antibiotic use, and elevate patient care in resource-limited regions.

## Introduction

Sepsis arises when an infection already present in the body triggers a widespread inflammatory response leading to rapid tissue damage, organ failure, and death if not treated promptly.^1^ Bacterial infections are the leading cause of sepsis.^2^ Zhang et al., have applied modelling to examine the burden in China and conclude that bloodstream infections (BSIs) will continue to exhibit the highest clinical burden, further complicated by antimicrobial resistance (AMR).^3^ Children, particularly in low- and middle-income countries (LMICs) most effected where poverty plays a major part in clinical outcome.^4^^,^^5^ However, data from LMICs is scant due to clinical and laboratory limitations as has been recently shown from the Kenyan survey.^6^

The “gold standards” for diagnosing infections and sepsis involves the detection and isolation of pathogens from sterile body fluid specimens using standard culture techniques. Blood culture (BC) systems, prevalent in high-income countries, are instrumental in this diagnostic process. These systems provide conditions for microorganism growth in blood culture bottles (BCBs) by maintaining constant temperature and agitation, and they continuously monitor for growth through changes detected by colourimetric or fluorescent CO_2_ sensors in the BCBs.^7^ Such systems have been the backbone of BC diagnostics but have their limitations in terms of sensitivity and time to positivity (TTP).^8^

However, the economic cost of BC systems presents a significant challenge in developing countries. In China, most BC instruments and corresponding BCBs are supplied by Becton Dickinson (BD) or bioMérieux. In many LMICs and regions, the high cost of BC instruments and BCBs make it challenging for hospitals to afford and implement diagnostic equipment to detect and manage sepsis. For countries that currently lack BC equipment, the introduction of affordable yet scientifically robust BC instruments could significantly enhance the diagnosis of sepsis across the “global South”, where for example, very few African microbiological laboratories are sufficiently equipped to diagnose sepsis.^9^ For hospitals already equipped with BD or bioMérieux systems, using compatible yet more affordable BCBs could greatly reduce hospital costs and national health budgets.^10^^,^^11^ Where BC diagnostics are lacking, treatment is via empirical antibiotics and efficacy is based on clinical response only, often resulting in inappropriate antibiotics being ministered for a protracted period of time.^11^, ^12^, ^13^ Furthermore, there is no microbiological information to aid infection control practices.^10^ Despite technological developments in detection, there is a lack of large-scale comparative data on BC instruments and BCBs.^11^, ^12^, ^13^ Therefore, we deployed a large prospective clinical study to: i, evaluate the performance of alternative BC instruments and BCBs (specifically, Meihua Medical [MH] and DL-Biotech [DL]); ii, the interchangeability of BC instruments and BCBs across different brands (specifically, MH or DL BCBs were analysed in BD or bioMérieux BC instruments); and iii, understand how such systems could address diagnostic sustainability in LMICs.

## Methods

### Study design

We conducted a nine-month evaluation of BC instruments and BCBs across clinical laboratories in five tertiary hospitals from September 2023 to June 2024. Permission to use the information from the medical records of the patients for research purposes was granted by the ethical committee of the Second Affiliated Hospital of Zhejiang University, School of Medicine (Number: 2024-0435). Personal patient details were not involved at any stage of the research, and study-specific sample numbers were used without any patient identifiers. To ensure the scientific robustness of the study and avoid potential interference from the spread of multidrug-resistant strains or overlapping patient populations within the same site, we selected hospitals of varying sizes and from different regions across Zhejiang province, China.

For the instrument evaluation, we selected Affiliated Jinhua Hospital of Zhejiang University School of Medicine (H1) and the Second Affiliated Hospital of Zhejiang University School of Medicine (H2). For the evaluation of BCBs, we selected the Fourth Affiliated Hospital of Zhejiang University School of Medicine (H3), Wenzhou Medical University Affiliated Dongyang Hospital (H4), and the First Affiliated Hospital of Zhejiang University School of Medicine (H5). H2 and H5, are the largest teaching hospitals in Zhejiang, and located in Hangzhou, the capital city of Zhejiang, while the other three hospitals are local hospitals located in Jinhua, Yiwu, and Dongyang, respectively.

**Fig. 1:**
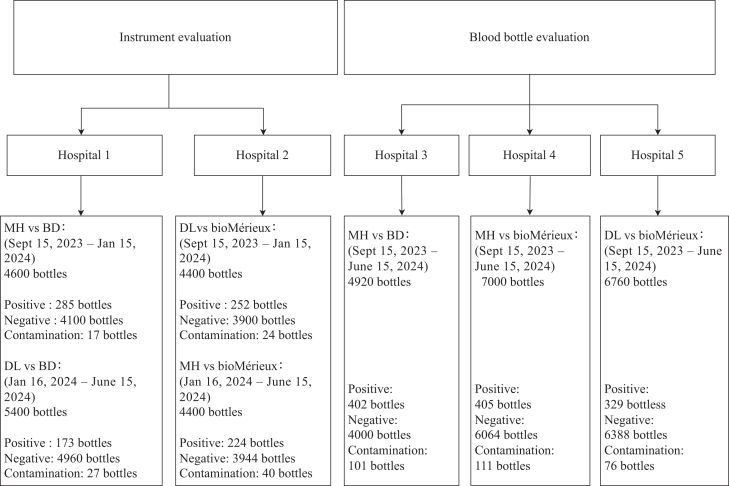
**Flowchart diagram of blood culture instruments and bottles evaluation in five hospitals.** Hospital 1, Affiliated Jinhua Hospital of Zhejiang University School of Medicine; Hospital 2, the Second Affiliated Hospital of Zhejiang University School of Medicine. Hospital 3, the Fourth Affiliated Hospital of Zhejiang University School of Medicine, Hospital 4, Wenzhou Medical University Affiliated Dongyang Hospital; Hospital 5, the First Affiliated Hospital of Zhejiang University School of Medicine.

After the initial four months of the study, we exchanged instruments between H1 and H2 to exam interchangeability and ease-of-use. In H1, we initially compared MH and BD, then switched to comparing DL with BD. For H2, we started with DL and bioMérieux and subsequently changed to MH and bioMérieux after four months. We conducted bottle comparisons at H3 [MH (Fluorescence) versus BD], H4 [MH (colourimetry) versus bioMérieux], and H5 (DL versus bioMérieux) (Fig. 1). During the observational clinical trial, each patient had blood samples collected using a dual-BCB method. Blood samples were not biased towards any one clinical sector and collected from various wards, including the intensive care unit (ICU), emergence intensive care unit (EICU), emergency department, etc. The clinical diagnosis of BSIs in each hospital was not altered throughout the study and neither was the aseptic taking of blood by local hospital staff. Where possible, blood was extracted prior to the administration of any antibiotics.

### Procedures

#### Performance verification

Before testing clinical samples, we conducted performance verification of the instruments at H1 and H2 by spiking BCBs. The TTP was chosen as the time from inoculation to when a positive result was detected. We selected seven different species of bacteria, including obligate aerobic bacteria (*Acinetobacter baumannii, Pseudomonas aeruginosa*), facultative anaerobic bacteria (*Escherichia coli, Klebsiella pneumoniae, Staphylococcus aureus*), fastidious bacteria (*Streptococcus pneumoniae, Haemophilus influenzae*), and one yeast (*Candida albicans*), comprising a total of 16 strains (one ATCC strain and one clinical isolate for each species). Verification of BC systems and BCBs were conducted in three parallel groups. Briefly, following established protocols,^14^^,^^15^ we diluted all overnight cultures to concentrations of 10^2^ Colony-forming unit (CFU)/mL and 10^1^ CFU/ml. We subsequently inoculated BCBs with 1 ml of each dilution into aerobic and anaerobic bottles from BD, bioMérieux, MH, and DL. Simultaneously, we performed colony counts (Figure S1) by plating onto appropriate media to determine the precise quantity of bacteria inoculated into the BCBs as previously described.^16^ Additionally, 8 ml of sheep blood was added to the *H. influenzae* BCBs.

For the bacterial species *S. aureus, E. coli, K. pneumoniae, S. pneumoniae*, and *H. influenzae*, each isolate was cultured simultaneously in both aerobic and anaerobic BCBs as previously described.^17^ Each bottle was processed in parallel on four automated systems: BD, DL, bioMérieux, and MH. For each bacterial species, three replicate measurements were obtained per bottle per instrument, resulting in six TTP values per instrument per species.

To assess differences in TTP for the same species across different instruments and between aerobic and anaerobic bottles, appropriate statistical methods were employed. First, the normality of the TTP data distribution was evaluated. Data conforming to a normal distribution were described using the mean plus or minus the standard deviation (mean ± SD), while data not meeting normality assumptions were reported using the median and interquartile range (median [IQR]). For normally distributed TTP data, the assumption of sphericity was subsequently tested. When the sphericity assumption was satisfied (*P* > 0.05), repeated measures analysis of variance (ANOVA) was used to assess intergroup differences. A *P*-value <0.05 was considered statistically significant. In cases where sphericity was violated (*P* < 0.05), the Greenhouse-Geisser corrected *P*-value was used to determine statistical significance.

For TTP data not following a normal distribution, the Friedman two-way ANOVA test was employed to assess intergroup differences, with *P*-value <0.05 indicating statistical significance. Where multiple comparisons were required, the Bonferroni correction was applied, and a *P*-value <0.05 was considered indicative of a significant difference between groups. For *P. aeruginosa, A. baumannii*, and *C. albicans*, only aerobic bottle experiments were conducted. Statistical analyses for these species followed the same procedures as described above. All statistical analyses were performed using SPSS software (IBM Corp., Armonk, NY, USA).

### Clinical sample

To complement the clinical evaluation, we first conducted a controlled laboratory-based performance assessment using artificially cultured strains. Each bacterial isolate was inoculated in triplicate into both aerobic and anaerobic BCBs from BD, bioMérieux, MH, and DL, and subsequently incubated on their respective instruments. This phase enabled a standardised comparison of TTP across systems under controlled conditions. However, due to the limited number of replicates (n = 3) and the use of laboratory strains, these results may not fully reflect the complexity of clinical infections, or the variability observed in real-world hospital settings. Therefore, we proceeded to conduct a clinical study using patient-derived blood samples to further evaluate system performance in practical diagnostic contexts.

Due to the nature for the study and the volumes of blood required, patient exclusion criteria included paediatrics and neonates. For blood samples, we collected two sets of BCs (each set consisting of one aerobic bottle and one anaerobic bottle) from each patient suspected of having a bloodstream infection. One set of blood was used to inoculate either DL or MH BCBs, and the other set used to inoculate BD or bioMérieux BCBs, with 8–10 ml of blood inoculated into each bottle. Blood was distributed equally by volume. All samples were immediately sent to the laboratory and loaded onto their respective BC instruments (Fig. 1) within 2 h. In hospitals evaluating instruments, MH or DL BCBs were placed into their corresponding MH or DL BC instruments (Fig. 1). In hospitals evaluating BCBs, MH or DL BCBs were placed in BD or bioMérieux BC instruments (Fig. 1). The incubation period for all four systems was set to a maximum of five days. Upon detecting a positive signal, we performed gram staining and simultaneously subcultured positive BCBs onto 5% sheep blood agar and chocolate agar media (Autobio Diagnostics Co., Ltd), as well as China blue agar media (Hangzhou Binhe Microorganism Reagent Co., Ltd) with incubation at 35 °C in a 5% CO_2_ environment for 18–24 h. Subsequent identification was carried out according to the standard procedures of each hospital. All five hospitals used MALDI-TOF MS for bacteria identification (Bruker for H1 and H2, Autobio for H3, bioMérieux for H4 and H5).

In our analysis of TTP of clinical samples, we specifically focused on clinically significant pathogens, including *E. coli* and ESKAPE pathogens. However, due to the limited number of *E. faecium* isolates, we included all detected *Enterococcus* spp. in our analysis. Additionally, in accordance with national guidelines, we excluded contaminated bottles from our calculations.^16^ These criteria include, (i) Only one of the four bottles is positive, and the bacteria are CoNS (coagulase-negative *Staphylococci*) or *Corynebacterium*, *Lactobacillus*, etc. was also excluded. (ii) 2/4 bottles are CoNS or *Corynebacterium*, *Lactobacillus*, etc., but the patient did not have a clinical presentation consistent with septicaemia.

Chi-square tests were employed to compare categorical variables such as positivity rates between MH and DL, and BD or bioMérieux BCBs, with Fisher's exact test used when expected frequencies were less than five. Detection results from individual clinical samples were collected from hospital records and reflect real-world outcomes based on routine diagnostic procedures. No experimental interventions were introduced, and each sample was tested only once; therefore, each case contributed a single, non-replicated data point. To assess differences in TTP across different instruments and aerobic BCBs from four BC systems, appropriate statistical analyses were conducted.

For TTP data conforming to a normal distribution, one-way ANOVA was used to evaluate intergroup differences. In cases where the data did not meet normality assumptions, the Kruskal–Wallis H test was employed as a non-parametric alternative. A *P*-value <0.05 was considered statistically significant. When multiple pairwise comparisons were required, the Bonferroni correction was applied, and a *P*-value <0.05 was used to determine statistical significance between groups. All statistical analyses were performed using SPSS software.

### Affordability questionnaire

A succinct and exploratory questionnaire was sent to our clinical colleagues in Nigeria, Sierra Leone, Ethiopia, and Pakistan who are clinically qualified and are fully cogent of the issues faced by patients in their countries (Table S6). The costs were given in local currency and covered to US dollars using Xe currency converter at the time of manuscript preparation. We also asked about the cost of standard treatment of sepsis–ampicillin and gentamicin is the WHO recommended first line treatment for sepsis, and ceftazidime and amikacin, is commonly used when clinical failure to the first line is evident.^2^

### Ethics approval

Permission to use the clinical information for research purposes was approved by the Ethics Committee of the Second Affiliated Hospital of Zhejiang University, School of Medicine (Approval Number: 2024-0435). All data were anonymised, and no personal identifiers were involved at any stage of the study.

### Role of the funding source

The funders of the study had no role in the study design, data collection, data analysis, data interpretation, or writing of the report. None of the four companies involved in the evaluation of blood culture systems (BD, bioMérieux, MH, and DL) participated in the study design, funding (including in-kind support), data collection, data analysis, interpretation, manuscript preparation, or the decision to submit the manuscript for publication.

## Results

### Performance validation and time to positivity

At bacterial concentrations of 10^1^ and 10^2^ CFU/ml, significant differences in TTP were observed among the four automated BD systems (MH, DL, BD, and bioMérieux), with variation depending on organism and bottle type.

At 10^1^ CFU/ml (Table S1), *S. aureus* showed significantly longer TTP on MH and bioMérieux compared to BD and DL, both at the instrument and aerobic bottle level. For *E. coli*, bioMérieux exhibited significantly prolonged TTP across instruments and aerobic bottles compared to the other three systems. *K. pneumoniae* showed longer TTP on bioMérieux than on BD and MH, while DL did not differ significantly from bioMérieux; in aerobic bottle comparisons, only bioMérieux's had significantly longer TTP. For *S. pneumoniae*, MH yielded significantly longer TTP than BD, while DL and bioMérieux values were intermediate with no statistically significant differences from either; similar trends were observed among aerobic bottles. For *H. influenzae*, MH showed significantly longer TTP than the other three instruments, consistent with the aerobic bottle results. For *P. aeruginosa*, bioMérieux demonstrated the longest TTP, significantly longer than DL and BD; DL had the shortest. *A. baumannii* showed a significant difference only between BD and DL. For *C. albicans*, DL exhibited the shortest TTP, significantly faster than the other three systems.

At 10^2^ CFU/ml (Table S1), *S. aureus* showed significantly shorter TTP on the DL system compared to the other instruments, while no difference was observed between bioMérieux and MH; similar trends were noted in aerobic bottles. For *E. coli* and *K. pneumoniae*, bioMérieux exhibited significantly prolonged TTP relative to the other systems, with consistent findings in aerobic bottles. *S. pneumoniae* demonstrated longer TTP on DL and MH than on BD and bioMérieux, while for *H. influenzae*, DL yielded the longest and BD the shortest TTP; both results were mirrored at the bottle level. For *P. aeruginosa*, bioMérieux had the longest TTP, significantly exceeding that of DL and BD. In *A. baumannii*, significant differences were observed only between MH and bioMérieux. For *C. albicans*, BD exhibited the longest and MH the shortest TTP in aerobic bottles.

In summary, bioMérieux consistently exhibited prolonged TTP for *E. coli*, *K. pneumoniae*, and *S. aureus*, while MH and DL showed delayed detection of fastidious organisms like *S. pneumoniae* and *H. influenzae*, consistent with findings from aerobic bottle comparisons.

### Blood culture instrument evaluation

After confirming the performance validation from spiked samples, we conducted a non-inferiority study using clinical blood samples from patients presenting with suspected BSIs. As described in the methods, we eliminated BC contaminants from the results. In H1 and H2, we analysed 1150 sets of samples for both MH and BD, 1350 sets for both DL and BD, 1100 sets for both MH and bioMérieux, and 1100 sets for both DL and bioMérieux (Fig. 1). Statistical analysis of all samples showed a high concordance across all groups with no significant differences. When calculated by BC bottle sets, the concordance rates between MH versus BD, DL versus BD, MH versus bioMérieux, and DL versus bioMérieux were 97.2%, 97.7%, 98.0%, and 96.5%, respectively (Table S2). When calculated by aerobic bottles only, the concordance rates for MH versus BD, DL versus BD, MH versus bioMérieux, and DL versus bioMérieux were 98.2%, 98.5%, 98.4%, and 97.2%, respectively (Table S3).

When comparing MH and BD, the number of detected bacterial isolates was 163 versus 122, respectively. In the comparison between MH and bioMérieux, the number of bacterial isolates was 116 versus 108, respectively. When comparing DL to BD, the number of positive isolates was 83 versus 90, respectively, while for DL versus bioMérieux, it was 116 versus 136 isolates, respectively. However, in the comparison between DL and bioMérieux, DL demonstrated an advantage in detecting rare pathogens, identifying one isolate of *Mycoplasma hominis* and two isolates of *Cryptococcus neoformans* (Figure S2B).

### Blood culture bottle evaluation

**Fig. 2:**
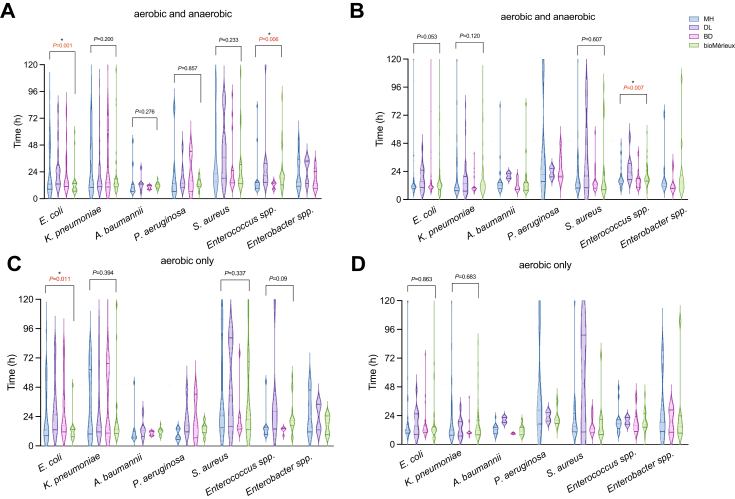
**Violin plots of TTP for *E. coli* and ESKAPE pathogens.** Panels (A) and (C) present the instrument evaluation data, calculated by sets (aerobic and anaerobic bottles) and aerobic bottles only, respectively. Panels (B) and (D) display the bottle evaluation data, calculated by sets and aerobic bottles only, respectively. The shape of each violin plot reflects the distribution of data (the width represents the kernel density), with the central dotted line indicating the median. The upper and lower dotted line of the violin represent the upper and lower quartiles, respectively. Group comparisons were performed using the nonparametric Kruskal–Wallis H test, with Bonferroni correction applied for multiple comparisons. Asterisks (∗) indicate statistically significant differences (*P* < 0.05), the absence of statistical results indicates an insufficient number of strains for analysis. Y-axis represents the TTP (in hours, with time intervals set at 24, 48, 72, 96 and 120 h). Data were derived from patient dual-BCB samples to assess the TTP using various instruments and bottles.

**Fig. 3:**
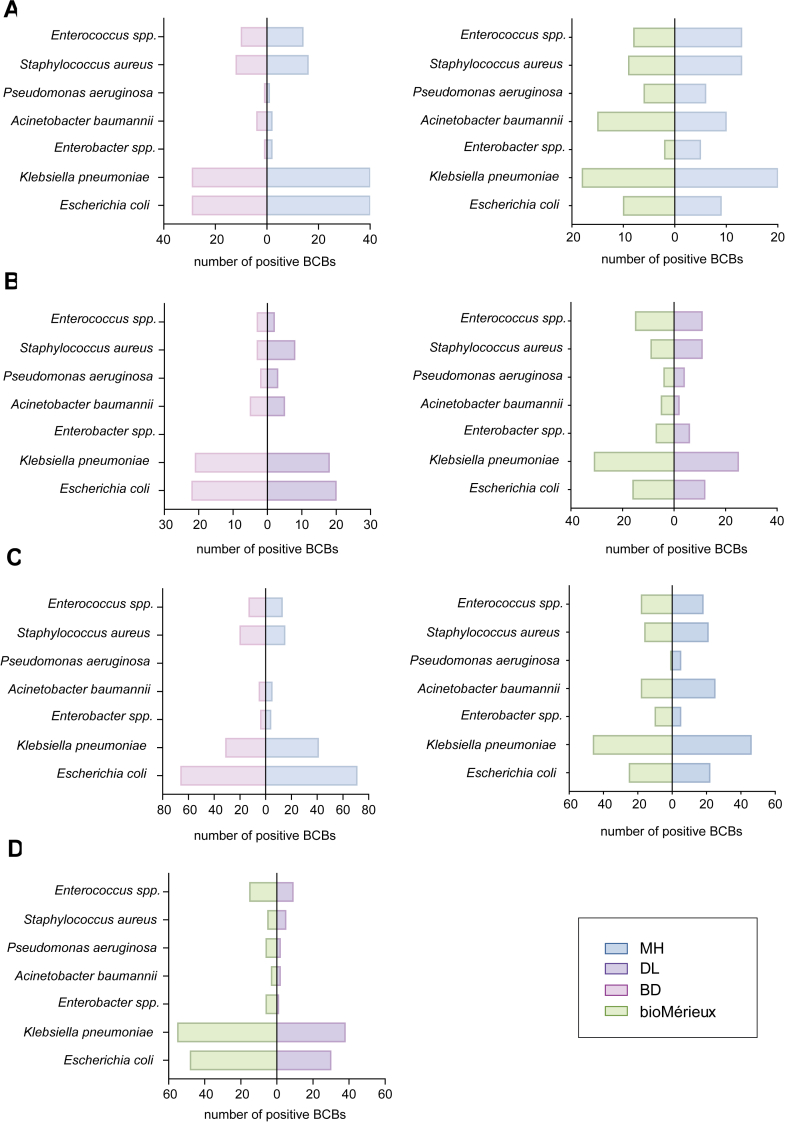
**Comparison of *E. coli* and ESKAPE Pathogens detected in the prospective clinical observational trial.** (A) MH versus BD and bioMérieux (instrument evaluation), (B) DL versus BD and bioMérieux (instrument evaluation), (C) MH versus BD and bioMérieux (bottle evaluation), (D) DL versus bioMérieux (bottle evaluation). X-axis represents the number of positive detections. Data were derived from patient dual-BCB samples to assess the detection capabilities of various instruments and bottles.

We simultaneously conducted bottle comparisons in three additional hospitals (H3, H4, and H5). Since MH uses both colourimetric and fluorescent bottles, our comparison included three groups: MH versus BD (fluorescent), MH versus bioMérieux (colourimetric), and DL versus bioMérieux. Throughout the study in H3, H4 and H5, we compared 1230, 1750, and 1690 BC bottle sets, respectively, with concordance rates of 97.3%, 98.4%, and 97.7% (Table S4). For aerobic bottles only, the concordance rates were 98.5%, 98.4%, and 98.2% (Table S5). Differences in consistency were observed only when comparing DL and bioMérieux bottles, whether assessed by bottle sets (*P* = 0.009) or by aerobic bottle only (*P* = 0.017). We analysed the distribution of positive isolates and the TTP for *E. coli* and ESKAPE (Fig. 2B, D, and 3C) and found that MH performed well with both colourimetric and fluorescent bottles, showing comparable results to BD and bioMérieux.

When comparing MH versus BD bottles, and MH versus bioMérieux bottles, 209 and 193, and 205 and 200 isolates were detected, respectively. However, MH was less effective than BD in detecting anaerobic bacteria (one isolate for MH versus five isolates for BD) (Figure S2C). DL showed a significantly decrease in detection rate when compared to bioMérieux, with 130 versus 199, respectively (Fig. 3D, Figure S2D). Despite DL's lower detection number, there was no significant difference in TTP for *E. coli* and ESKAPE pathogens among each of the four brands, except for *Enterococcus* spp., where the TTP calculated by bottle sets showed a significant difference (*P* = 0.007), with DL having a longer TTP compared to MH. For *Enterococcus* spp., the shortest median TTP was observed with BD (15.18 h), followed by MH (16.08 h) and bioMérieux (18.20 h), with DL having the longest mean TTP (23.43 h). In the evaluation of *Enterococcus* spp., BD and MH consistently exhibited the shortest TTP. Specifically, MH demonstrated the fastest detection in the instrument comparison, whilst BD showed the shortest median TTP in the bottle comparison.

### Analysis of discrepant results

Discrepant results occur when one instrument or when one BCB brand detects bacteria while the comparator does not or detects different bacteria. In our comparisons, MH exhibited the highest positivity rate among the instruments evaluated; specifically, there were 40 discrepant cultures between MH and BD, with MH detecting 25 isolates and BD detecting 15. In comparisons between MH and bioMérieux, there were 24 discrepant cultures, with MH identifying 13 isolates and bioMérieux identifying 11 isolates. During these comparisons, six isolates from the MH versus BD group and three isolates from the MH versus bioMérieux group gave a TTP exceeding 48 h. In comparisons involving DL, there were 35 discrepant cultures with BD, with DL detecting only 14 isolates compared to 21 by BD. Similarly, there were 43 discrepant cultures with bioMérieux, where DL identified 17 isolates versus 26 by bioMérieux. Among these, nine isolates from the DL versus BD group, and seven isolates from the DL versus bioMérieux group, gave a TTP over 48 h (Figure S3A). Analysis of the discrepant cultures showed that DL had inferior anaerobe detection capabilities compared to BD and bioMérieux. The specific distribution of inconsistent positive results in the instrument comparison is shown in Figure S4A–D.

When comparing BCBs, MH detected 38 discrepant cultures compared to BD and 36 compared to bioMérieux, with eight and four isolates, respectively, giving a TTP exceeding 48 h (Figure S3B). Of the 38 discrepant cultures in the MH versus BD comparison, MH detected 22 isolates, while BD detected 16. In the MH versus bioMérieux comparison, both systems detected 18 isolates each. Additionally, MH demonstrated fewer anaerobe detection, while BD detected four anaerobic isolates. The detailed distribution of discrepant cultures in bottle comparisons is shown in Figure S4E–G. For DL versus bioMérieux BCBs, DL BCBs exhibit weaker detection capability in the bioMérieux instruments, with 15 isolates of *E. coli* and *K. pneumoniae* not being detected. However, the mean TTP for these bacteria in bioMérieux instruments (30.3 h for *E coli* and 24.1 h for *K. pneumoniae*) were longer when compared to the mean TTP observed in the performance validation (12–14 h for *E. coli* and 18–20 h for *K. pneumoniae*).

In many LMICs, diagnostics invariably costs considerably more than antibiotics, and therefore, even for serious infections such as sepsis, BCs are rarely routinely done, resulting in the empirical use of antibiotics.^11^, ^12^, ^13^ As part of our LMICs network, we conducted a questionnaire to understand the cost of antibiotics (ampicillin [AMP] plus gentamicin [GEN], and ceftazidime [CTZ]) commonly used to treat sepsis (AwARE list) (Table S7). Furthermore, we undertook a cost assessment of the four BC systems including maintenance and support (Table 1). Commercially, DL outperforms MH in terms of international sales and after-sales service; however, MH is not as well established as DL, BD or bioMérieux. It is noteworthy DL requires the use of its proprietary bottles together with the DL instrument whereas as MH is more flexible as their bottles can be used with other manufacturers’ instruments.^18^ The cost of MH and DL instruments and bottles, presented in Figure S5, are significantly less than those of BD or bioMérieux (Table 1).

**Table 1:** Details of selected blood culture systems for evaluation study.

Brand	BD BACTEC FX 240	BioMer BacT/ALERT 3D 200	Meihua BC40/120	DL-Biotech BT-48/60^c^
**Blood culture instruments**				
Capacity	240 bottles	200 bottles	40/120 bottles	48 bottles/60 bottles
Dimensions (W x D x H)	67.5 cm × 58.5 cm × 39.1 cm	35.6 cm × 61.7 cm x 91.4 cm	BC40: 50.0 cm × 55.0 cm x 31.5 cm BC120: 51 cm × 55 cm x 78 cm	54 cm × 37.6 cm x 36.1 cm
Weight	31.8 kg	57.2 kg	BC40/120: 31/55 kg	24 kg
Power consumption	100–240 VAC ± 10%	100/120 V or 220/240 V	220 V	220 V ± 22 V
Detection	Fluorescence	Colourimetry	Fluorescence/Colourimetry	Colourimetry
Normal operating conditions	18.0–30.0 °C; 25%–80% humidity; no condensation	10.0–30.0 °C; 10%–90% humidity; no condensation	5.0–35.0 °C; ≤80% humidity. 76–106 KPa atmospheric pressure	10–30 °C; ≤80% humidity. 76–106 KPa atmospheric pressure
Reading interval	10 min	10 min	10 min	10 min
Price of instrument ($)^a^	Approx. 58,000	Approx. 48,000	6800/14,000	6500/8000
Max warranty	3 years	3 years	12 months	18 months
**Bottles**				
Type	BD BACTEC™ Plus Aerobic/F Culture Vials, BD BACTEC™ Lytic/10 Anaerobic/F Culture Vials	BacT/ALERT FA Plus, BacT/ALERT® SN	Fluorescence: Aerobic Culture Bottle (FA), Anaerobic Culture Bottle (FN) Colorimetry: Aerobic Culture Vials (CFA), Anaerobic Culture Vials (CFN)	Aerobic Blood Culture Bottle, Anaerobic Blood Culture Bottle
Storage temperature	2∼25 °C, avoid light	15∼30 °C, avoid light	2∼36 °C, avoid light	15∼30 °C, avoid light
Shelf-life	9 months	12 months	12 months	12 months
Pack	50 bottles/box	100 bottles/box	25/50/100/200 bottles/box	20/40 bottles/box
Optimal blood volume	8–10 ml	10 ml	8–10 ml	10 ml
Broth volume	Plus Aerobic/F Culture Vials: 25 ml Lytic/10 Anaerobic/F Culture Vials: 40 ml	BacT/ALERT FA Plus: 30 ml BacT/ALERT® SN: 40 ml	FA/FN: 30 ml CFA/CFN: 30 ml	Aerobic Blood Culture Bottle: 30 ml Anaerobic Blood Culture Bottle: 30 ml
Price of bottle ($)^b^	5–6	5–6	1.5	1.4

a Price quoted was from quotes and purchase of instruments during BARNARDS in 2021–2022.

b Note: The bottle price is based on a minimum order of 5000 bottles. Prices were taken from BARNARDS and varied from country–country.

c Note: Recommended to use the brand's own bottles with their instruments for optimal performance and compatibility.

## Discussion

BCs are, despite their limitations, a critical diagnostic tool for detecting BSIs, which remain a significant cause of mortality in low-income countries.^8^ BD and bioMérieux were among the earliest brands to exported BC bottles and instruments, and both brands quickly gained international traction due to their advanced diagnostic capabilities, reliability and successful marketing. BD's BACTEC system and bioMérieux's BacT/ALERT systems are widely used across hospitals world-wide, offering robust performance in detecting a wide range of pathogens.^16^^,^^17^ The development of alternative BC systems in China began with the production of BCBs compatible with BD and bioMérieux instruments. For instance, companies such as Zhuhai DL Biotech., Zhuhai MH Medical Technology, and Autobio Diagnostics registered their BC bottles with the National Medical Products Administration (NMPA) in the mid-2010s.^19^ Subsequently, these companies developed and registered fully automated BC instruments reducing dependency on imported products and making significant advancements in BC technology. MH and DL are well-regarded in the Chinese market for their reproducible performance and reliability. Previous studies have highlighted their comparable detection capabilities to imported bottles; however, these studies have focused on simulated (e.g. spiked) samples only.^1^^,^^15^, ^16^, ^17^, ^18^ In contrast, our study not only validated these findings with spiked samples, but also conducted a large-scale, multi-arm prospective study of both the BCBs and BC instruments using hospital blood samples.

Our study highlights the competitiveness of alternative BC systems to established “gold-standard” bottle and instruments (BD and bioMérieux). Although statistically significant differences in TTP were observed during performance validation, the main discrepancies for the systems MH and DL were noted in the detection of fastidious organisms, specifically *H. influenzae* and *S. pneumoniae*. However, these findings were likely influenced by the small number of replicates, which limit their generalisability.

In contrast, real-world clinical data offered a more representative assessment. TTP results for common pathogens obtained using MH and DL were broadly comparable to those from BD and bioMérieux. Although the detection time for *H. influenzae* in MH bottles was longer (BD demonstrating the shortest TTP), it did not detract from the overall performance of MH. In the instrument evaluation using clinical samples, two isolates of *H. influenzae* were cultured from MH bottles with TTP of 18.7 h and 30.2 h, respectively, and one isolate was cultured from the DL bottle with a TTP of 14.6 h, whereas no *H. influenzae* were isolated using BD. The performance of DL for spiked samples was similar to that reported by Carrasco et al.^18^ Due to the small number of isolates, statistical analysis was not possible. Similarly, the TTP in performance verification for *E. coli* and *K. pneumoniae* were different to the clinical samples, whether instrument or bottle comparisons. These results are not unexpected and indicate the variations in clinical sample comparisons due to differences in bacterial load between patients and potential differences in blood volume distribution. Further analysis of real-world clinical samples revealed that significant TTP differences among systems were only seen for *Enterococcus* spp. and *E. coli*, with DL consistently showing longer TTP than the other platforms. In these cases, MH demonstrated performance comparable to BD and bioMérieux; however, was not as sensitive at culturing anaerobes. For other ESKAPE pathogens, no significant differences in TTP were observed, suggesting overall equivalence across systems in routine clinical detection.

Taken together, our findings demonstrate that despite some species-specific differences, particularly for *E.* spp. and *E. coli*, MH and DL systems are generally comparable to gold-standard instruments in terms of TTP performance in clinical settings. These alternative systems may therefore serve as reliable diagnostic options, especially in LMICs where resource constraints limit access to molecular diagnostics and culture remains the primary method for bloodstream infection detection.^6^^,^^11^^,^^13^^,^^19^^,^^20^

Of note, in the clinical observational trial, MH bottles exhibited higher detection rates for *E. coli* and ESKAPE pathogens than the other three brands which is an important finding given their association with sepsis in LMICs.^20^, ^21^, ^22^ In contrast, DL demonstrated slightly higher detection rates for rare pathogens such as *M. hominis* and *C. neoformans*. Notably, while significant differences in consistency were observed between DL and bioMérieux bottles, the overall TTP for key pathogens such as *E. coli* and *K. pneumoniae*-the two most frequently isolated bacteria in BSIs in China^23^ and among the top five leading pathogens in sepsis-related deaths remained comparable across all systems.^22^^,^^24^ These findings underscore the sensitivity of these alternative bottles/systems compared to BD and bioMérieux.

The 2024 UN declaration on AMR has been recently ratified by member states including its proposed targets—reduction in mortality and antibiotic use is a long-term strategy to tackle global AMR.^25^ Additionally, the International Health Partnership aims to expand the coverage of essential interventions and improve health outcomes by harmonising donor efforts and aligning aid with national priorities.^26^^,^^27^ Further, digital healthcare solutions, supported by the WHO, are being developed to enhance healthcare delivery in LMICs regions, underscoring the importance of strengthening healthcare systems and addressing global health inequity.^28^ Many laboratories in LMICs, and particularly in Africa, do not have laboratory capacity to diagnose critical infections such as sepsis where access, supply chain and cost remain critical factors.^13^^,^^28^ Accordingly, antibiotics are given empirically and patient management is often devoid of laboratory support and microbiological guidance. The overwhelming mitigating factor across LMICs public health is the cost of instruments and, in particular consumables, and the deferment of cost to patients who cannot afford either the laboratory tests or appropriate antibiotics mitigating appropriate clinical care. Our questionnaire in LMICs hospitals would indicate that BC diagnosis needs to be approximately $5 or below for this to be implemented across most LMICs public health sectors. Our assessment confirms the recent investigation by Hyland et al., that examined the cost of BCBs in LMICs and also concluded that for many patient groups the cost is prohibitive.^28^^,^^29^ Our study reveals that BCBs from DL and MH are substantially less expensive than their international counterparts. This cost reduction can result in significant savings for healthcare institutions, allowing for more extensive testing, better resource allocation and bridging the global “south-north” divide.^6^ The compatibility of MH bottles with existing BD and bioMérieux systems also provides additional flexibility, enabling hospitals to transition to more cost-effective solutions and contributes to the sustainability of healthcare improvements in LMICs.^6^^,^^30^^,^^31^ Whilst the data from our study is encouraging, rigorous quality control and adherence to regulatory standards is essential for any diagnostic system, and further analysis on MH and DL BC bottles and instruments are required across diverse clinical settings prior to international approval.^32^^,^^33^

Despite this being the first large clinical observational study to compare alternative bottles and instruments to “gold standards”, our study has several limitations. Firstly, the focus on a limited number of tertiary hospitals may not fully represent all clinical settings. While the study includes a substantial number of samples, further research involving a larger and more diverse patient population across different regions and countries would help confirm our findings. Secondly, our study evaluated the performance based on TTP and detection rates, while other factors such as ease of use and user satisfaction were not extensively evaluated. Furthermore, robustness and durability should be examined. Thirdly, we did not perform blind subculturing after a 5-day negative BC result which may have detected low-level or slow-growing organisms with extended incubation, potentially affecting the overall accuracy and reliability of our findings.

In conclusion, our study demonstrates that alternative BCBs/BC instruments can provide a robust and cost-effective alternative to “gold-standard” systems. These affordable and cost-effective alternatives could facilitate the strengthening of healthcare infrastructures in LMICs and improve health outcomes where cost, access and supply chains are an ongoing concern. The cost-effectiveness and compatibility of these systems make them a practical choice for resource-limited settings, potentially leading to enhanced diagnostic capabilities for critical infections and better patient management in LMICs public hosptials.^34^

## Contributors

YH, RZ and TRW conceived and designed the study. YF, ZY, ZZ, QS, SW, YJ, XQ, YZ, YM, LB, WB, XX, LS, YXZ, YC, HZ, JC, HM and LH undertook the study and data collection and analysis. CC, SW, YH and RZ undertook data analysis. TRW, RZ, YH, and YZ wrote the majority of the manuscript, and took the decision to submit the article. All authors had direct access and verified the underlying data in this article.

## Data sharing statement

The majority of the data described in this manuscript is accessible in the Supplementary information. Raw data sets that are not listed in the article can be made available on request to YH and RZ. There are no restrictions on data sharing.

## Declaration of interests

Yanyan Hu reports no relationships/activities/interests from third parties.

Yinfei Fang reports no relationships/activities/interests from third parties.

Zelin Yan reports no relationships/activities/interests from third parties.

Zhiqiang Zhu reports no relationships/activities/interests from third parties.

Qiaoling Sun reports no relationships/activities/interests from third parties.

Sipei Wang reports no relationships/activities/interests from third parties.

Yanyan Jiang reports no relationships/activities/interests from third parties.

Xinhua Qiang reports no relationships/activities/interests from third parties.

Chang Cai reports no relationships/activities/interests from third parties.

Yanyan Zhu reports no relationships/activities/interests from third parties.

Yongjun Ma reports no relationships/activities/interests from third parties.

Lihong Bu reports no relationships/activities/interests from third parties.

Wenzi Bi reports no relationships/activities/interests from third parties.

Xiaoping Xia reports no relationships/activities/interests from third parties.

Lingbin Shu reports no relationships/activities/interests from third parties.

Yangxiao Zhou reports no relationships/activities/interests from third parties.

Yunxiang Cai reports no relationships/activities/interests from third parties.

Hongwei Zhou reports no relationships/activities/interests from third parties.

Jiachang Cai reports no relationships/activities/interests from third parties.

Hanqiang Miao reports no relationships/activities/interests from third parties.

Lin Huang reports no relationships/activities/interests from third parties.

Yuqing Zhou reports no relationships/activities/interests from third parties.

Shaolin Wang reports no relationships/activities/interests from third parties.

Rong Zhang reports no relationships/activities/interests from third parties.

Timothy R. Walsh reports no relationships/activities/interests from third parties.

## Appendix B Supplementary data

Supplementary Figures and Tables

Chinese summary
