# Supplementary material for: Evaluation of alternative blood culture systems in detecting pathogenic bacteria: a multi-site, observational prospective study
Source: Lancet Reg Health West Pac. 2026 Feb 2;67:101806. doi: 10.1016/j.lanwpc.2026.101806 (PMC12887412; doi:10.1016/j.lanwpc.2026.101806)
Supplement: Supplementary Figures and Tables [file mmc1.pdf]

Supplementary Appendix

Table S1. Time to positivity (TTP; mean ± SD) for different bacterial species cultured in aerobic and anaerobic bottles across four instruments
Table S2. Consistency rate calculated by bottle sets in instrument evaluation
Table S3. Consistency rate calculated by aerobic bottles in instrument evaluation
Table S4. Consistency rate calculated by bottle sets in blood bottle evaluation
Table S5. Consistency rate calculated by aerobic bottles in blood bottle evaluation
Table S6. Questionnaire on cost of antibiotics and diagnostics, and cost deferment to the patient
Table S7. Cost of antibiotics versus blood culture diagnostics in Sierra Leone, Nigeria, Pakistan and Ethiopia

Figure S1. Flowchart of performance validation.
Figure S2. Bubble plot of bacterial distribution detected by various brands during the comparison of instruments and bottles.
Figure S3. Radar plots depicting the number of inconsistent strains detected at different time points (12h, 24h, 36h, 48h, and ≥72h).
Figure S4. Symmetric bar chart of the distribution of inconsistently bacteria.
Figure S5. Photographs of domestic instruments and bottles.

Table S1. Time to positivity (TTP; mean ± SD) for different bacterial species cultured in aerobic and anaerobic bottles across four instruments

	10^1 aerobic and anaerobic bottles				10^1 aerobic bottles			
	BD	DL	bioMérieux	MH	BD	DL	bioMérieux	MH
*S. aureus**	15.0 (1.6)	12.6 (0.6)	18.9 (2.9)*	16.3 (4.0)	16.3 (1.0)	12.4 (0.7)	20.9 (2.7)*	20.0 (0.9)
*E. coli**	9.1 (1.0)	10.2 (0.6)	12.2 (0.9)*	10.1 (0.7)	9.9 (0.7)	10.4 (0.6)	12.8 (0.5)*	10.1 (0.8)
*K. pneumoniae**	10.4 (0.7)	11.1 (0.5)	13.1 (0.7)*	10.9 (0.9)	10.8 (0.6)	10.9 (0.3)	13.1 (0.6)*	10.2 (0.5)
*S. pneumoniae**	22.2 (1.5)	29.7 (6.4)*	26.4 (5.2)	29.3 (4.9)	22.2 (1.6)	25.9 (6.2)	23.9 (2.5)	26.0 (2.0)*
*H. influenzae**	17.5 (2.2)	29.0 (2.7)	31.6 (6.6)	37.6 (2.7)*	16.0 (1.7)	28.7 (3.2)	25.6 (1.5)	36.1 (2.2)*
*P. aeruginosa**	NA	NA	NA	NA	15.6 (1.0)	14.7 (0.8)	19.6 (1.2)†	17.0 (1.2)
*A. baumannii**	NA	NA	NA	NA	15.3 (2.2) *	12.2 (1.1)	13.6 (0.7)	13.7 (1.6)
*C. albicans**	NA	NA	NA	NA	34.8 (4.5)	25.2 (2.6)	36.7 (4.3)	41.6 (1.6)*
	10^2 aerobic and anaerobic bottles				10^2 aerobic bottles			
	BD	DL	bioMérieux	MH	BD	DL	bioMérieux	MH
*S. aureus**	13.0 (1.7)	11.3 (0.5)	16.6 (3.2)*	15.6 (2.9)	14.1 (0.94)	11.3 (0.5)	19.2 (2.1)*	18.3 (0.8)
*E. coli**	8.2 (0.9)	9.0 (0.6)	10.8 (0.4)*	9.0 (0.5)	8.9 (0.5)	9.4 (0.6)	10.8 (0.4)*	8.9 (0.5)
*K. pneumoniae**	8.9 (0.4)	9.9 (0.5)	11.8 (0.5)*	9.7 (0.5)	8.9 (0.4)	9.6 (0.6)	11.5 (0.4)*	9.6 (0.4)
*S. pneumoniae**	18.5 (0.9)	24.9 (1.9)	21.2 (1.5)	26.8 (4.8)*	19.0 (0.8)	24.3 (2.2)*	20.8 (1.8)	22.6 (2.0)
*H. influenzae**	16.9 (2.0)	26.0 (3.0)*	22.1 (3.4)	23.7 (3.6)	16.0 (2.1)	28.5 (2.0)*	19.1 (0.7)	20.8 (1.6)
*P. aeruginosa**	13.7 (1.1)	13.7 (1.0)	17.7 (1.2)†	14.4 (0.8)	13.7 (1.1)	13.7 (1.0)	17.7 (1.2)†	14.4 (0.8)
*A. baumannii**	13.5 (2.4)	11.4 (1.5)	12.4 (0.4)*	11.0 (1.1)	13.5 (2.4)*	11.4 (1.5)	12.4 (0.4)	11.0 (1.1)
*C. albicans**	28.5 (2.9)*	23.3 (1.3)	22.9 (1.7)	19.1 (1.3)	28.5 (2.9)*	23.3 (1.3)	22.9 (1.7)	19.1 (1.3)

NA, not applicable.

An asterisk (*) indicates a statistically significant difference in TTP among the four blood culture systems. This symbol is also placed at the data point corresponding to the system with the longest TTP when the difference is significant. Statistical analysis was performed using repeated measures ANOVA.

A dagger symbol (†) indicates that the data do not follow a normal distribution. However, due to the limited number of observations ($n = 3$), the values in parentheses are still reported as standard deviations (SD).

Table S2. Consistency rate calculated by bottle sets in instrument evaluation

	H1				H2			
	MH versus BD		DL versus BD		MH versus bioMérieux		DL versus bioMérieux	
	MH	BD	DL	BD	MH	bioMérieux	DL	bioMérieux
Total positive bottle sets	95	87	65	71	80	79	80	90
Consistent positive	75	75	53	53	69	69	66	66
Inconsistent positive	20	12	12	18	11	10	14	24
Contaminants bottle sets	10	7	10	17	13	11	10	11
Negative bottle sets	1045	1056	1275	1262	1007	1010	1010	999
Total bottle sets	1150		1350		1100		1100	
Overall consistency	97·2%		97·7%		98·0%		96·5%	
P value #	0·589		0·660		1·000		0·472	

Comparisons of consistency rates were conducted using the Chi-square test.

Table S3. Consistency rate calculated by aerobic bottles in instrument evaluation

	H1				H2			
	MH versus BD		DL versus BD		MH versus bioMérieux		DL versus bioMérieux	
	MH	BD	DL	BD	MH	bioMérieux	DL	bioMérieux
Total positive bottles	80	76	44	56	71	74	69	77
Consistent positive	68	68	40	40	64	64	58	58
Inconsistent positive	12	8	4	16	7	10	11	19
Contaminants bottles	8	7	8	14	9	10	8	11
Negative bottle sets	1062	1067	1298	1280	1020	1016	1023	1012
Total bottle sets	1150		1350		1100		1100	
Overall consistency	98·2%		98·5%		98·4%		97·2%	
P value #	0·804		0·262		0·864		0·549	

Comparisons of consistency rates were conducted using the Chi-square test

Table S4. Consistency rate calculated by bottle sets in blood bottle evaluation

	H3		H4		H5	
	MH versus BD		MH versus bioMérieux		DL versus bioMérieux	
	MH	BD	MH	bioMérieux	DL	bioMérieux
Total positive bottle sets	136	129	132	134	89	127
Consistent positive	113	113	120	120	89	89
Inconsistent positive	19	12	12	14	0	38
Contaminants bottle sets	54	32	56	42	14	50
Negative bottle sets	1040	1069	1562	1574	1587	1513
Total bottle sets	1230		1750		1690	
Overall consistency	97·3%		98·4%		97·7%	
P value #	0·696		1·000		0·009	

Comparisons of consistency rates were conducted using the Chi-square test.

Table S5. Consistency rate calculated by aerobic bottles in blood bottle evaluation

	H3		H4		H5	
	MH versus BD		MH versus bioMérieux		DL versus bioMérieux	
	MH	BD	MH	bioMérieux	DL	bioMérieux
Total positive bottles	117	115	119	126	63	93
Consistent positive	108	108	109	109	63	63
Inconsistent positive	11	7	10	17	0	30
Contaminants bottles	44	24	44	26	9	44
Negative bottles sets	1069	1091	1587	1598	1618	1553
Total bottle sets	1230		1750		1690	
Overall consistency	98·5%		98·4%		98·2%	
P value #	0·954		0·691		0·017	

Comparisons of consistency rates were conducted using the Chi-square test.

Table S6. Questionnaire on cost of antibiotics and diagnostics, and cost deferment to the patient.

Questionnaire on cost of antibiotics and diagnostics, and cost deferment to the patient.
1. How much is the combination of ampicillin-gentamicin/day/patient?
2. How much is the combination of ceftazidime-amikacin/day/patient?
3. How much would a blood culture need to be for introduction into a public hospital to the point where it becomes affordable to all?
4. What cost for the above is born by the patient?
5. What extend is the cost of diagnostics covered by the insurance and who has access to national/local insurance schemes.

The questions were asked of our clinical and academic leads who are part of our clinical burden studies. Our LMIC colleagues only operate in public health institutions and are very knowledgeable on the cost of antibiotics and diagnostics, as well as costs deferred to the patient. The combination of ampicillin and gentamicin is the WHO recommended first line treatment for sepsis, and ceftazidime and amikacin, is, in our experience commonly used when clinical failure to the first line is evident.

Table S7. Antibiotic costs for the treatment of sepsis compared to BC diagnostics in LMICs

	**Sierra Leone**	**[a]Nigeria**	**Ethiopia**	**Pakistan**
i.v. AMP + GEN	$5-6	$1-2	$4	$3
i.v. CTZ + AMK	$22	$2.5-6	$8 (CTZ only)	$4
Maximum price for BC	$7	<$5	$6*	$3**
Diagnostic costs borne by patients?	Hospitals bear most of the costs for diagnostics but financial resources are limited	100% except for the few with insurance	* If available in hospital BC would be free but needs to be sub $8 for hospital support. Cost of private BC is $15.	**$8 for patients paying for private services. $3 would be the estimated maximum cost if borne by the patient
Antibiotic costs borne by patients?	AMP and GEN are free but hospital stockouts are common and antibiotics are then bought privately from local pharmacies	100% except for the few with insurance	AMP-GEN free<br><br>AMK is often not available	100% free to admitted patients until stockouts

BC, blood culture; LMICs, low- and middle- income countries; AMP, Ampicillin; GEN, Gentamicin; CTZ, Ceftazidime; AMK, Amikacin. Cost of therapy is given as an average of the combination/day.
Maximum price for BCs is that that can be afforded by either the hospital and/or patient in a public healthcare setting.

[a]The number cited represents a mean from five hospitals/regions in Nigeria including state and federal health institutions.

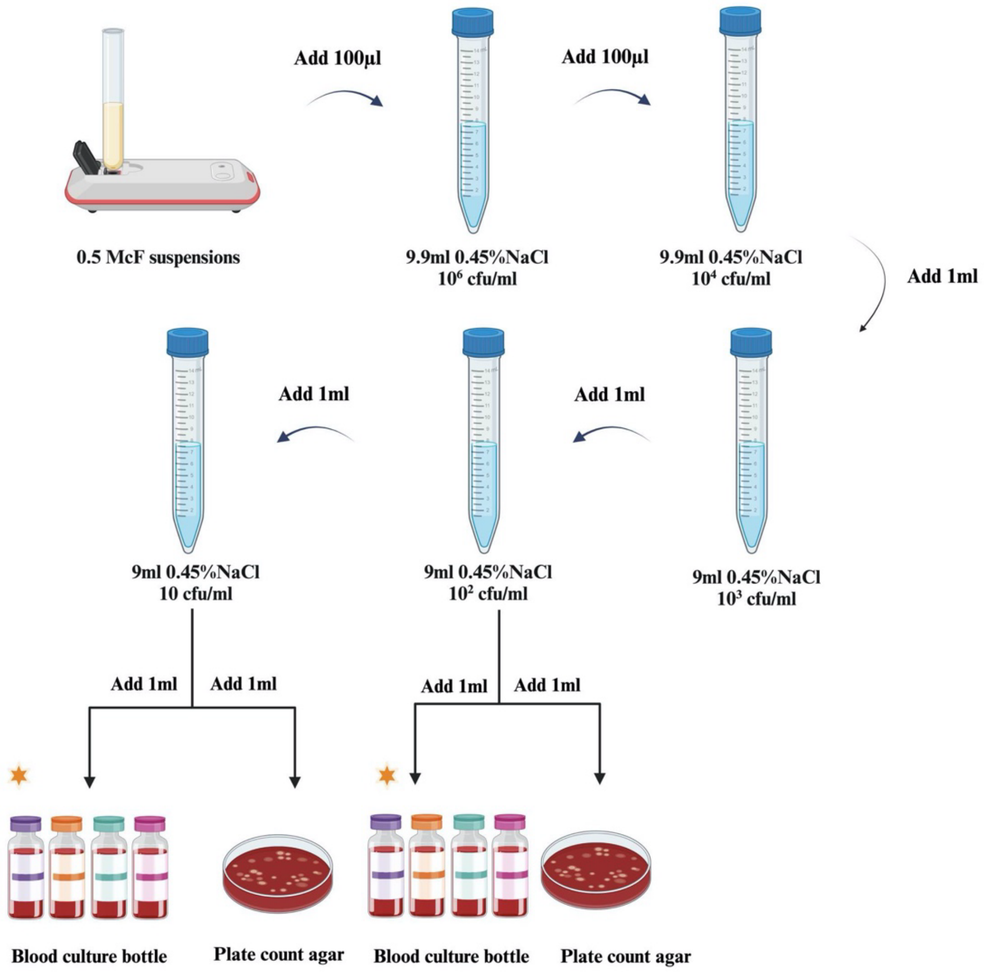

Figure S1. Flowchart of performance validation. * In the validation of *Haemophilus influenzae*, 5 mL of 5% sheep blood was added.

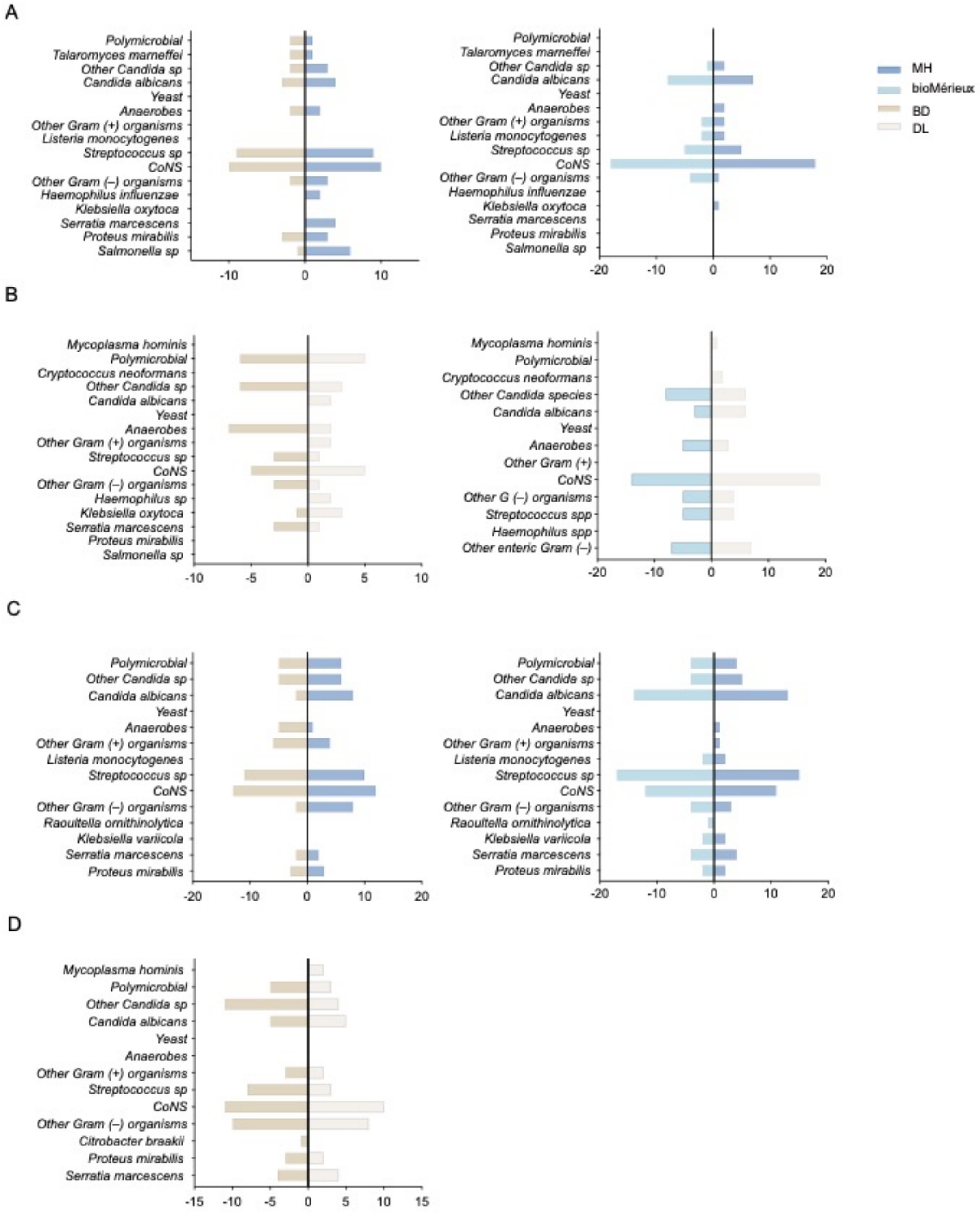

Figure S2. Distribution of bacteria, including those beyond *E. coli* and ESKAPE pathogens, detected by various brands during the comparison of instruments and bottles. (A) Comparison of MH with BD and bioMérieux in instrument comparison, (B) Comparison of DL with BD and bioMérieux in instrument comparison, (C) Comparison of MH with BD and bioMérieux in bottle comparison, (D) Comparison of DL with bioMérieux in bottle comparison. CoNS, Coagulase-Negative *Staphylococci.* The CoNS shown here are those identified as true pathogens responsible for bloodstream infections.

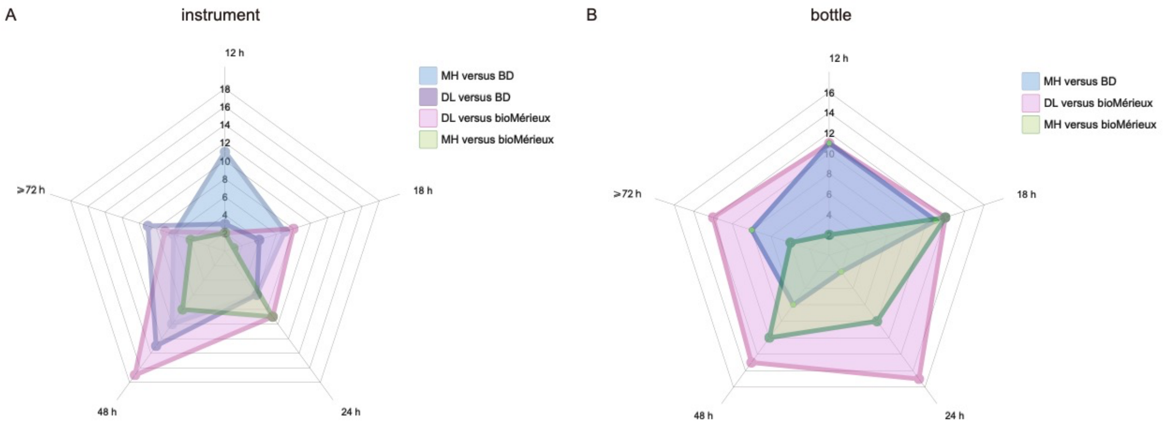

Figure S3. Radar plots depicting the number of inconsistent strains detected at different time points (12h, 24h, 36h, 48h, and ≥72h). (A) Instrument evaluation, (B) Bottle evaluation. Each radar plot represents the number of discrepant strains detected at five TTP intervals: ≤12 h, 12–18 h, 18–24 h, 24–48 h, and ≥72 h. The concentric layers correspond to the number of isolates detected within each time range.

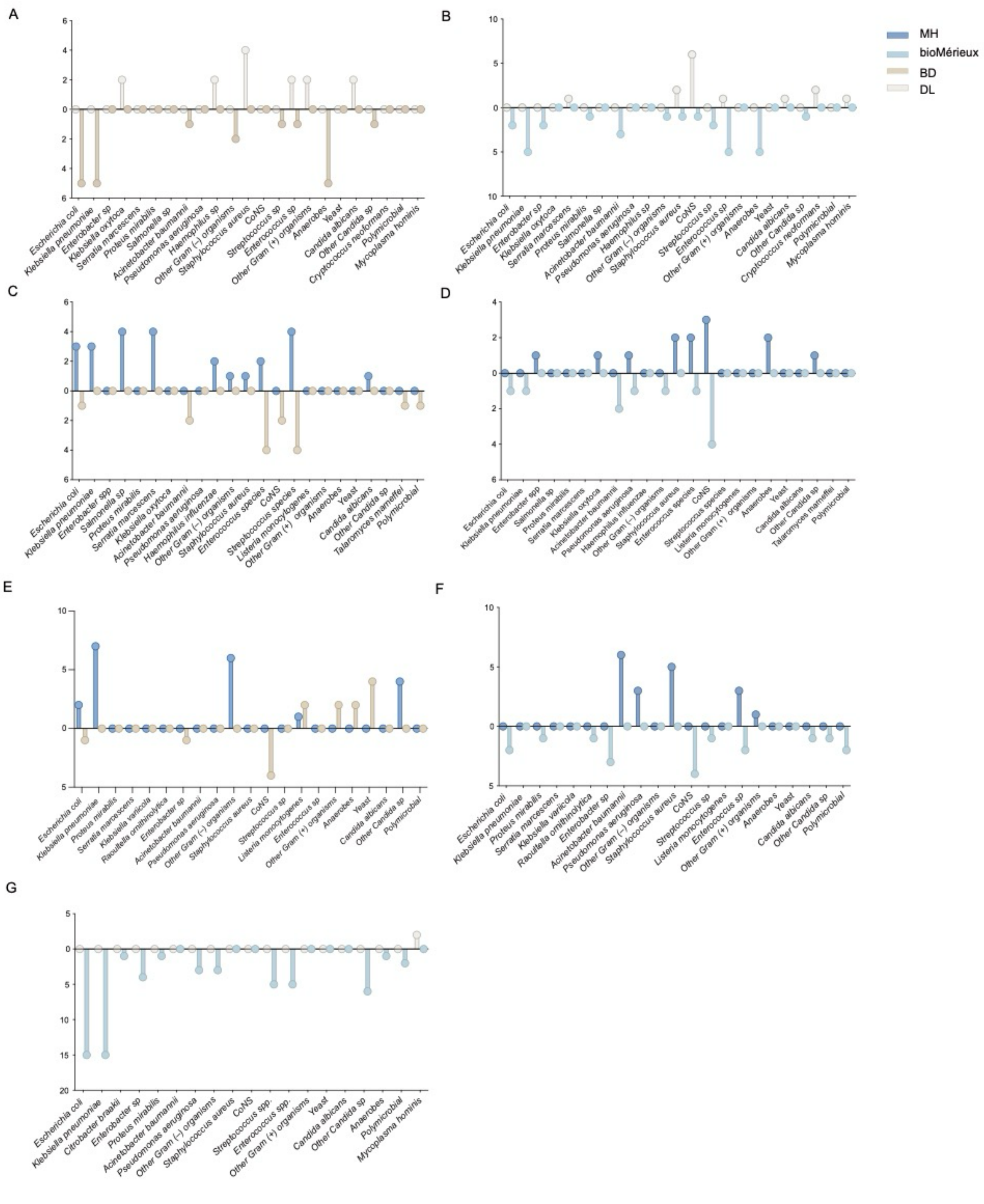

Figure S4. Symmetric bar chart of the distribution of inconsistently bacteria. (A) Instrument evaluation of MH versus BD, (B) Instrument evaluation of MH versus bioMérieux, (C) Instrument evaluation of DL versus BD, (D) Instrument evaluation of DL versus bioMérieux, (E) Bottle evaluation of MH versus BD, (F) Bottle evaluation of MH versus bioMérieux, (G) Bottle evaluation of DL versus bioMérieux. The x-axis shows the bacterial species involved in discrepant results, while the y-axis indicates how many isolates of each were detected. For clarity, some species are grouped together when necessary.

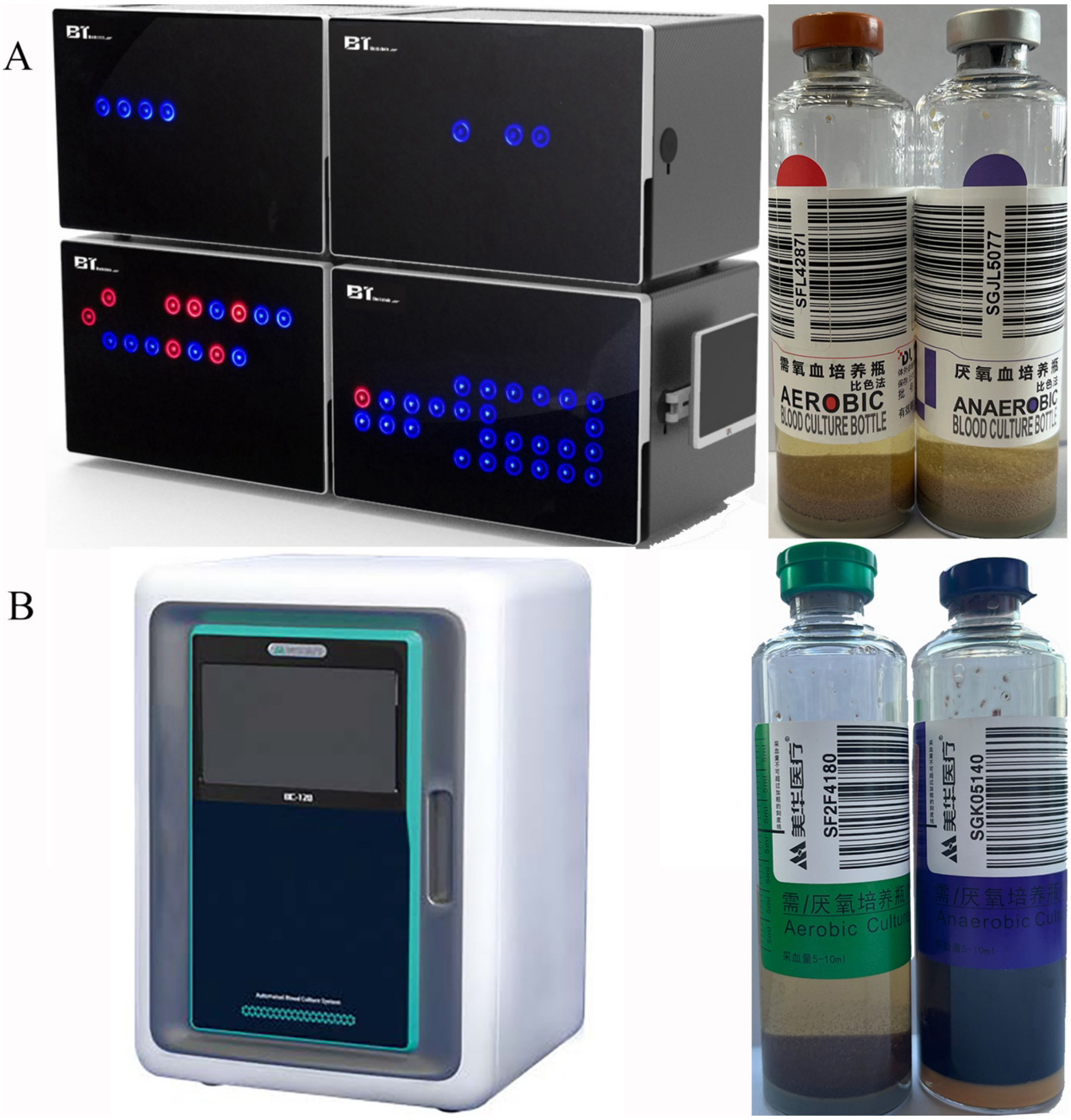

Figure S5. Photographs of alternative instruments and bottles. (A) DL instrument and bottles, (B) MH BC120 instrument and colorimetric bottles.
