# Supplementary material for: Evaluation of alternative blood culture systems in detecting pathogenic bacteria: a multi-site, observational prospective study
Source: Lancet Reg Health West Pac. 2026 Feb 2;67:101806. doi: 10.1016/j.lanwpc.2026.101806 (PMC12887412; doi:10.1016/j.lanwpc.2026.101806)
Supplement: Chinese summary [file mmc2.docx]

摘要

背景：

脓毒症是一种由感染引起的严重且危及生命的全身炎症反应，其发病率持续上升并伴随显著的病死率。血培养（BC）系统是脓毒症诊断的关键工具，但其高昂成本限制了在低收入地区的推广应用。本研究旨在评估低成本血培养仪器及培养瓶的性能，并分析其与国际公认标准系统的兼容性。

方法：

2023 年 9 月至 2024 年 6 月，我们在中国浙江省五家三级医院开展了一项为期九个月的多中心评估研究。两种国产替代性血培养仪器（美华医疗 [MH] 与迪尔生物 [DL]）及其配套血培养瓶（BCBs）与国际主流系统（bioMérieux 与 Becton Dickinson [BD]）进行比较。在处理临床样本前，通过向培养瓶中接种大肠埃希菌（E. coli）及ESKAPE 菌群，检测各系统的阳性检出时间（TTP）及性能表现。研究期间共检测37,480 个BCBs。

结果：

DL 和 MH 仪器在常见细菌的 TTP 表现方面总体良好。在非劣效性观察分析中，仪器间的一致性分别为：MH 对 BD 为 97.2%，DL 对 BD 为 97.7%，MH 对bioMérieux 为 98.0%，DL 对 bioMérieux 为 96.5%。在灵敏度方面，MH 检测到的菌株数量多于 BD（163 对 122）和 bioMérieux（116 对 108）。在血培养瓶比较中，MH 与BD 的阳性培养数为 209 对 193，MH 与 bioMérieux 为 205 对 200。然而，MH 在厌氧菌检出方面略低于 BD。DL 的总体检出率低于 bioMérieux（130 对 199），但在 E. coli 和 ESKAPE 菌株的 TTP 上表现相似，仅在肠球菌属上表现出较长的 TTP。

结论：

我们对 DL 和 MH 血培养系统的评估表明，其整体性能可与 BD 和 bioMérieux 系统相媲美。鉴于其性能表现与成本优势，这些低成本血培养仪器及培养瓶在低收入和中等收入国家的临床应用中应得到更多重视，同时有助于强化合理的抗菌药物管理。

基金：

本研究由中国国家重点研发计划（编号：2022YFD1800400）及香港特别行政区政府研究资助局主题研究计划（T11-104/22-R）资助。

关键词：

脓毒症；血培养；阳性检出时间；低收入与中等收入国家

This translation in Chinese was submitted by the authors and we reproduce it as supplied. It has not been peer reviewed. Our editorial processes have only been applied to the original abstract in English, which should serve as reference for this manuscript.
